# Exploring temporal varying demographic and economic disparities in COVID-19 infections in four U.S. areas: based on OLS, GWR, and random forest models

**DOI:** 10.1007/s43762-021-00028-5

**Published:** 2021-12-04

**Authors:** Junfeng Jiao, Yefu Chen, Amin Azimian

**Affiliations:** Urban Information Lab, The School of Architecture, the University of Texas at Austin, Austin, TX 78705 USA

**Keywords:** COVID-19, Geographically weighted regression, Demographic and economic disparities, Neighborhood, GIS, Random forest

## Abstract

Although studies have previously investigated the spatial factors of COVID-19, most of them were conducted at a low resolution and chose to limit their study areas to high-density urbanized regions. Hence, this study aims to investigate the economic-demographic disparities in COVID-19 infections and their spatial-temporal patterns in areas with different population densities in the United States. In particular, we examined the relationships between demographic and economic factors and COVID-19 density using ordinary least squares, geographically weighted regression analyses, and random forest based on zip code-level data of four regions in the United States. Our results indicated that the demographic and economic disparities are significant. Moreover, several areas with disadvantaged groups were found to be at high risk of COVID19 infection, and their infection risk changed at different pandemic periods. The findings of this study can contribute to the planning of public health services, such as the adoption of smarter and comprehensive policies for allocating economic recovery resources and vaccines during a public health crisis.

## Introduction

Originally appearing at the end of 2019, COVID-19 is a new viral disease caused by a highly transmissible coronavirus known as SARS-CoV-2. Shortly after its discovery, the World Health Organization (WHO) declared COVID-19 a global pandemic on March 11, 2020 (WHO, n.d.). As of October 17, 2021, the number of confirmed cases in the U.S. has reached 45 million, with more than 747 thousand deaths recorded (WorldMeter, 2021).

As we are at the post-peak stage of this pandemic, many researchers have attempted to analyze the socio-economic disparities during the COVID19 pandemic (Credit, 2020; Liu et al., 2020a; Liu et al., 2020b; Quinn & Kumar, 2014; Sannigrahi et al., 2020; Wu & Zhang, 2021). It stands to reason that COVID-19 can affect vulnerable communities disproportionately. Previous studies concluded that people of color had experienced heightened COVID-19 infections and more deaths on average than white individuals (APM Research Lab, 2021). Besides, spatial heterogeneities were significant in COVID-19 infections (Hou et al., 2021; Thomas et al., 2020). The neighborhoods where COVID-19 gathered were likely where the vulnerable population lived (Hong et al., 2021).

Notably, there are three research gaps among previous studies. First, studies on COVID-19 predictors have largely been cross-sectional so far, yet it is possible that predictors change over time (CDC, 2021; Gray, 2021). The vulnerable groups may not have enough opportunities to get tests at the early stage of the pandemic, so that the number can be underestimated (Abdalla et al., 2021). With the increasing of responses to the pandemic, vulnerable groups can take the tests, and the number can be relatively more accurate at the post-peak stage. Understanding the spatial and temporal patterns of COVID-19 and sociodemographic and economic disparities in COVID-19 infections is vitally essential to combat and prevent outbreaks effectively and equitably allocate vaccines (Jiao & Azimian, 2021). Also, the differences between the situation at the early and post-peak stages can present the disparities in medical resources.

Secondly, previous studies used county−/city- level data (Liu et al., 2020a; Mollalo et al., 2020; Wu & Zhang, 2021). There lacks the precision necessary to discern spatial associations given the unequal distribution of populations within counties. However, it is hard to access the higher resolution data that allows for neighborhood-level analysis since most agencies only publish county-level data.

Last but not least, recent studies focused on urbanized regions with high population densities (Brakefield et al., 2021; Chen et al., 2020; Hu & Chen, 2021). Few looked at low-density areas since the spread of COVID-19 in low-density areas is anticipated to be slow (Wong & Li, 2020). However, it does not mean that COVID-19 transmission in low-density areas is mild. Low-density areas are often neglected by local authorities in disease control because of a variety of reasons. Given the low medical care accessibility in these areas, vulnerable groups face a higher risk when the pandemic attacks. Besides, according to a report of John Hopkins University, more than half of areas with 100 deaths per million people are low-density areas (CSSE, 2021). Therefore, it is imperative to place equal attention on disease prevention in urban and suburban areas, and it is worth comparing the differences between regions.

In terms of these research gaps, this study investigates demographic and economic disparities in COVID-19 infections over time and compares the difference between areas. Also, this study applies multiple models to empirically examine which models perform well in analyzing the relationship between demographic and economic factors and infections. We sought to answer three research questions:

- What are the demographic and economic disparities in COVID-19 infections?
- What are the changes in disparities when choosing different regions?
- Do these disparities change over time?

Research into these questions can contribute to planning public health services, such as new interventions to mitigate future health crises, equitable allocations of vaccines, and economic reliefs to help vulnerable groups recovery from the pandemic. With the released of high-solution (e.g., zip code level) data, this study can provide a micro-level perspective of COVID-19 patterns, which can help implement plans to help the most vulnerable groups suffering the most. We also explored these disparities from three types of models, ordinary least squares regression, geographically weighted regression, and random forest and compared the differences. This paper is organized as follows. First, we introduced the measurements and the primary method in this study. Next, we presented the results to the above research questions. Then, we discussed the summary of the main findings and research and policy implications. At last, we conclude this study.

## Data and methods

### Study area

**Fig. 1:**
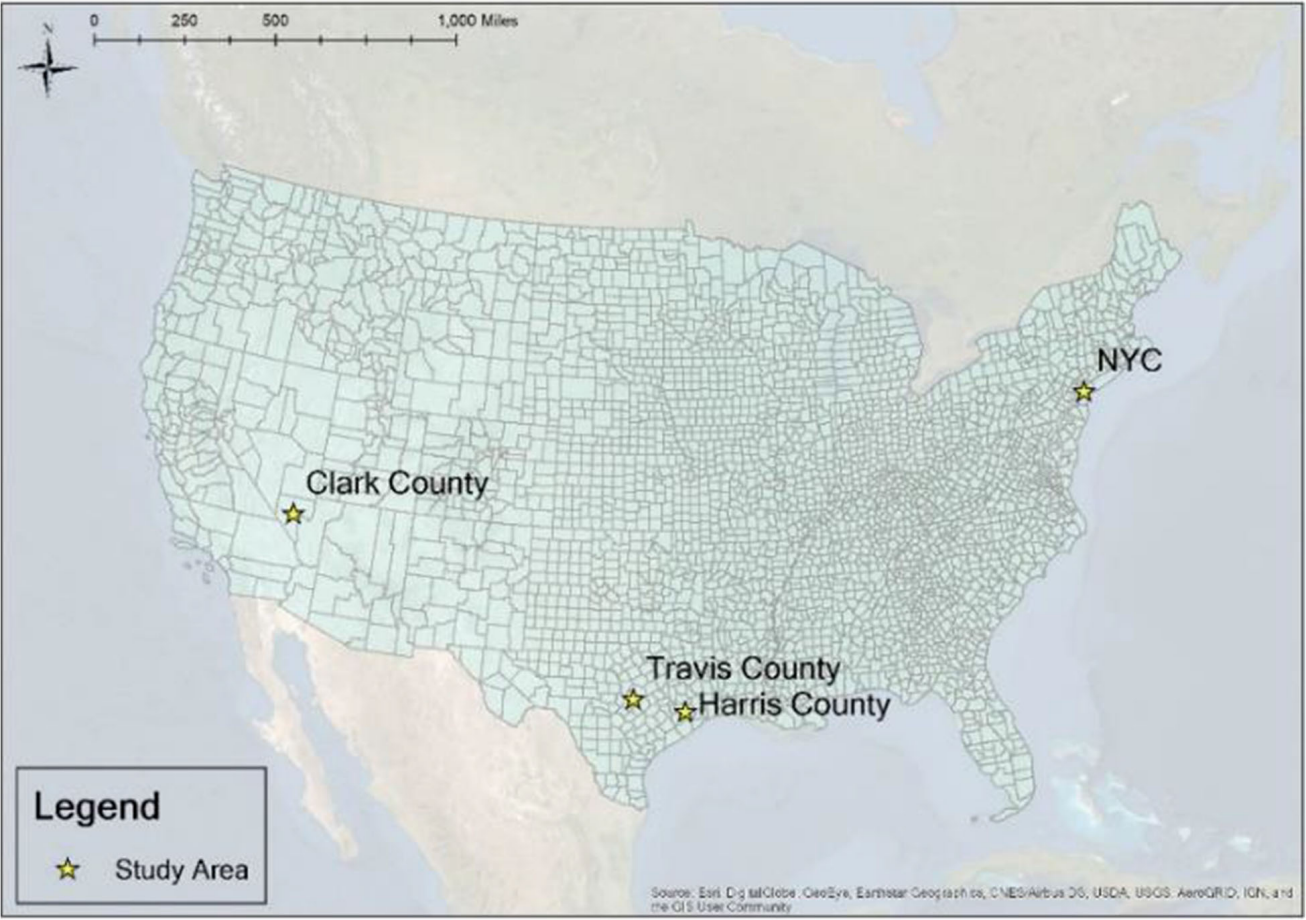
Study Area

Considering data accessibility, we selected four regions with different population densities as the study area. They are Travis County, Harris and Fort Bend counties, Clark County, and New York City (Fig. 1). The detailed descriptions of these samples are:

- Travis County (1300 people per square mile): Travis County locates in the center of Texas. It is where the capital of Texas locates, and the location of high-tech companies’ headquarters.
- Harris and Fort Bend Counties (2067 people per square mile): Harris County and Fort Bend County are the dominant part of the Houston Metropolitan Area. It is where manufacturing and logistic industries gather.
- Clark County (247 people per square mile): Clark County locates in the south and is the most populated county of Nevada. Since Las Vegas is the county seat, Clark County is famous for traveling, exhibitions, and gambling.
- New York City (29,302 people per square mile): New York City (NYC) is the most populated city around the U.S. It is an international metropolis with great influence in the economy, business, finance, media, politics, education, and entertainment.

Considering the population density, these samples can roughly present disparities in low-density areas, high-density areas, and densest area. We defined Clark County as the low-density area, Travis County and Harris and Fort Bend Counties as the high-density aeras, and NYC as the densest area based on the average metro population density in the U.S. (283 people per square mile) (Florida, 2012).

### Variables

The raw dataset included the African Americans rate, Hispanic Americans rate, older adult (65 years and older) rate, working from home rate, commuting through public transit rate, education attainment, and median distance traveled. After preliminary data preparation, we found that the rates of older adults (65+) and rates of people working from home were not suitable to be inputted since the VIF values with others were high (7.5). We dropped both these variables since GWR is sensitive to multicollinearity (ArcGIS, n.d.). Table 1 provides descriptions of the final dataset.

**Table 1:** Statistical descriptions of variables

Source	Variable Name	Date	Description	Harris and Fort Bend (***n*** = 163) Mean (S.D.)	Travis (***n*** = 56) Mean/S.D.	Clark (***n*** = 71) Mean (S.D.)	NYC (***n*** = 195) Mean (S.D.)
Local departments of health	*Case density*	June 5, 2020	Number of confirmed COVID-19 cases per 1000 people	3.132 (2.081)	2.629 (2.438)	2.694 (1.648)	6.492 (4.529)
		January 30, 2021		73.004 (32.265)	56.394 (85.178)	90.725 (48.907)	119.973 (78.685)
2018 American Community Survey	*Poverty*	2018	Predicted proportion of household in poverty within each zip code area	0.127 (0.084)	0.082 (0.068)	0.025 (0.016)	0.030 (0.025)
	*African American*	2018	Predicted proportion of African American within each zip code area	0.193 (0.161)	0.076 (0.069)	0.099 (0.076)	0.212 (0.244)
	*Hispanic American*	2018	Predicted proportion of Hispanic within each zip code area	0.390 (0.216)	0.303 (0.205)	0.264 (0.154)	0.260 (0.192)
	*Education*	2018	Predicted proportion of highest education attainment higher than college within each zip code area	0.074 (0.037)	0.100 (0.158)	0.040 (0.057)	0.054 (0.021)
	*Public transit*	2018	Predicted proportion of commuting through public transit within each zip code area	0.023 (0.022)	0.026 (0.029)	0.031 (0.035)	0.524 (0.160)
SafeGraph	*Distance traveled*	May 30, 2020, and December 7, 2021	Median distance (1000 m) traveled from the home	31.42 (13.40)	32.08 (15.42)	51.68 (31.13)	31.52 (28.12)
				41.81 (25.61)	39.88 (23.83)	63.71 (42.73)	48.43 (27.00)

Note: The study area included the administrative boundaries. The border areas were integrated as one if the zip codes were the same

**Fig. 2:**
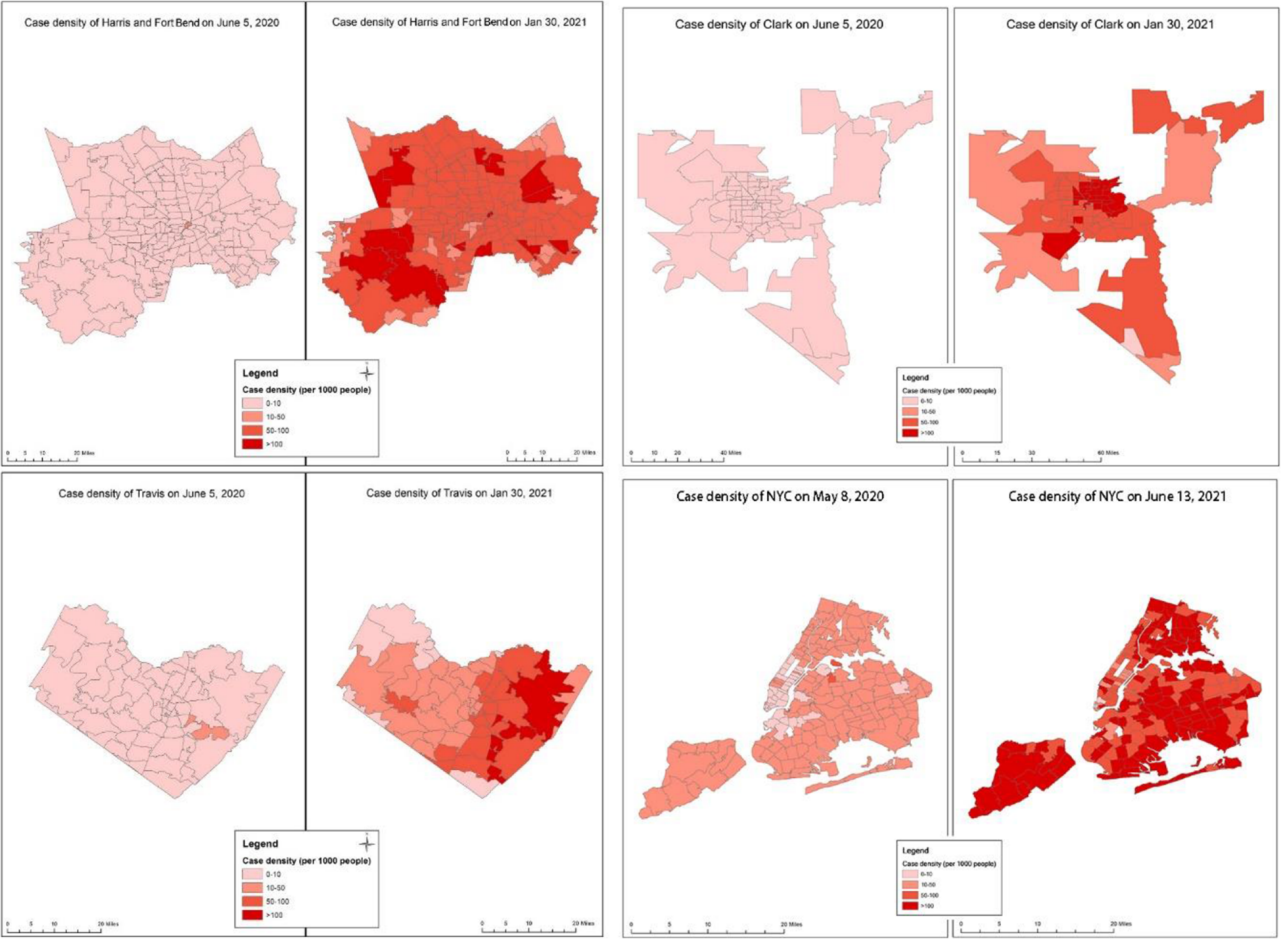
COVID-19 case density (per 1000 people) by zip code areas at the beginning and at the post-peak stages

The outcome variable is COVID-19 case density. We obtained the number of COVID-19 cases by zip code from local departments of health (City of Austin, n.d.; County, n.d.; Fort Bend County, n.d.; Harris County, n.d.; NYC Health, n.d.). Then, we captured the data in Clark County, Travis County, and Harris and Fort Bend Counties on June 5, 2020, as the “beginning” stage, and January 30, 2021, as the “post-peak” stage. However, NYC does not archive data at this period. We captured the NYC data on May 8, 2020, and June 13, 2021, instead. To normalize the COVID-19 cases by population, we calculated the density of COVID-19 cases, which refers to the number of confirmed COVID-19 cases per 1000 people, as the outcome variable in this study. Figure 2 presents the spatial distribution of COVID-19 case density in the study area.

Socio-demographic variables were considered as explanatory variables the final dataset. They were retrieved from the 2018 American Community Survey (ACS) at the Census block group level. The reason for focusing on African Americans and Hispanic Americans is that they have higher rates of hospitalizations and death than the white only during the pandemic (Qeadan et al., 2021; Tirupathi et al., 2020). Also, they were delayed receiving vaccines than the white only and presented mistrust of these vaccines (Kricorian & Turner, 2021). Considering that they occupy a large part (> 10%) of the total population, it is worth focusing on the disparities in COVID-19 among African Americans and Hispanic Americans.

Two travel behavior variables, the proportion of commuting through public transit and median distance traveled were captured at the Census block group level from ACS 2018 and SafeGraph (SafeGraph, n.d.). We captured the median distance traveled at the beginning period and post-peak period to measure the real-time travel activities.

Notably, spatial mismatches existed between the outcome variables (at the zip code level), the explanatory variables (at the census block group level), and the geographic boundaries of postal areas differed from those of census block groups. We integrated all explanatory variables into the zip code level based on spatial weights, assuming that population/workers/households were equally distributed.

### Methods

We applied multiple approaches to address the research questions, ordinary least squares regression (OLS), geographically weighted regression (GWR), and random forest (RF). OLS is used for fitting linear models. It finds values that minimize the sum of the squares of the difference between the actual value and the model estimate and use them as the coefficients to measure the impacts of explanatory variables. The equation for OLS is as follows:

$$ {Y}_t={\beta}_{0t}+{\beta}_{it}\ast {X}_{it}+\varepsilon $$

where *Y*_*t*_ is the COVID-19 case density, *β*_0*t*_ is the intercept of the model, *X*_*i*_ refers the *i*^*th*^ explanatory variable, *β*_*i*_ refers to the related coefficients, and *ε* is a random error term. *t* refers to the timestamp of the data.

Notably, OLS cannot test for spatial effects among observations and disregards spatial autocorrelation, while previous studies have indicated that COVID-19 outbreaks are spatially correlated (Mollalo et al., 2020). We applied GWR to measure the demographic and economic disparities in COVID-19 infections in terms of the spatial impacts. This method can deal with spatial autocorrelations and measure various coefficients from observations instead of estimating average coefficients in OLS models (Brunsdon et al., 1996; Goovaerts, 2008). We first applied Moran’s I to measure spatial autocorrelation. If the spatial autocorrelations were significant, we used GWR to determine the impacts of explanatory variables.

Recently, machine learning approaches have been popular in social science. After comparing the performance of several supervised learning algorithms, we chose RF to measure the demographic and economic disparities. RF is a supervised learning algorithm, which is an integrated learning algorithm based on the decision tree. It is simple and easy to implement, but it shows great performance in regression. Compared with traditional statistical models, the RF can judge the importance of features and the mutual influence between different features, which is not easy to overfit (Iwendi et al., 2020; Wang et al., 2020).

To sum up, we applied three models to measure the demographic and economic disparities in COVID-19 infections at different periods. We developed and ran the OLS and RF models through “statsmodels” and “sklearn” packages in Python 3.7 and applied GWR models in ArcGIS 10.6. For GWR models, we set the optimal bandwidth as the adaptive kernel and Akaike information criterion. The resulting R^2^ value was used to measure the model performance.

## Results

### OLS results

Table 2 presents the results of OLS models. Focus on the high-density areas at first. In the Harris and Fort Bend Counties region, the percentage of households in poverty and the percentage of African Americans played a significantly positive role in predicting COVID-19 case density at the beginning of the pandemic. At the post-peak stage, the percentages of African Americans and Hispanic Americans were significantly correlated with COVID-19 case density. These results indicate that residents who live in vulnerable communities, high rates of poverty minorities, are likely to experience a heightened risk of infections both during peak and post-peak stages. Also, the racial and ethnic groups were affected by the pandemic change over time. African Americans were the group most vulnerable at the beginning of the pandemic, and both American Africans and Hispanic Americans were significantly vulnerable at the post-peak stage. This claim is consistent with previous studies (Despres, 2021; Vasquez Reyes, 2020).

**Table 2:** OLS results

	***Dependent variable:*** Case density							
	Harris and Fort Bend 2020	Harris and Fort Bend 2021	Travis 2020	Travis 2021	Clark 2020	Clark 2021	NYC, 2020	NYC 2021
	(1)	(2)	(3)	(4)	(5)	(6)	(7)	(8)
Poverty	7.164** (3.257)	−64.654 (52.056)	4.994 (6.927)	522.208* (271.373)	26.404 (17.798)	164.940 (328.570)	−71.478*** (25.881)	390.224 (488.226)
African American	3.638*** (1.108)	46.138** (17.719)	5.721 (5.092)	− 325.611 (199.484)	3.123 (1.878)	−25.626 (34.396)	11.514*** (2.271)	45.154 (43.095)
Hispanic American	−0.370 (1.229)	70.016*** (19.649)	5.314** (2.160)	141.195 (84.603)	1.755 (1.333)	157.575*** (24.259)	20.031*** (3.022)	178.539*** (3.045)
Education	0.476 (4.100)	−29.749 (65.544)	−1.221 (1.848)	0.850 (72.410)	−4.745* (2.428)	725.282*** (44.285)	−16.758 (25.881)	−84.894 (25.900)
Public transit	−5.208 (6.982)	− 134.586 (111.607)	9.132 (10.286)	− 746.368* (402.973)	5.614 (7.779)	− 231.391 (142.156)	− 15.240*** (3.996)	−205.610*** (4.010)
Distance traveled	0.109 (0.115)	−1.858 (1.835)	−0.081 (0.176)	5.726 (6.909)	− 0.118*** (0.022)	−0.0732 (0.205)	0.139 (0.190)	14.386*** (3.745)
Constant	1.403** (0.646)	56.085*** (10.319)	0.316 (0.673)	−3.123 (26.356)	2.238*** (0.441)	26.757*** (6.745)	19.459*** (2.447)	106.377** (47.701)
Observations	163	163	56	56	71	71	195	195
R^2^	0.200	0.151	0.485	0.339	0.650	0.864	0.281	0.415

*Note:* ^*^p < 0.1; ^**^p < 0.05; ^***^p < 0.01; Stand errors are in parentheses

The situation in Travis County was different. Poverty was a critical factor in predicting the spread of COVID-19 at the post-peak stage in Travis County, although there was no significant correlation with this variable at the beginning of the pandemic. Surprisingly, public transit use was not significantly correlated with COVID-19 case density at the beginning of the pandemic, yet the coefficient is significantly negative at the post-peak stage. These results showed that the groups most vulnerable to COVID-19 infection changed over time in Travis County, from Hispanic Americans at the beginning of the pandemic to households in poverty during the post-peak stage. Public transit use might not promote the spread of COVID-19 at the post-peak stage.

Look at the situations in the low-density case. At the beginning stage, COVID-19 case density was significantly correlated with educational attainment and distance traveled. It means that communities with more educated residents and less travel had fewer infections than others. At the post-peak stage, neighborhoods with a higher rate of Hispanic Americans were likely to have more infections. Notably, Hispanic Americans have higher rates of hospitalizations and death (Contreras, 2021). Besides, we found that the coefficient of educational attainment turned positive. This finding is against with a claim that educational attainment decreases the risk of COVID-19 severity (Yoshikawa & Asaba, 2021).

The situation in the densest area is worth noting. First, the coefficients of rates of African Americans and Hispanic Americans were significantly positive at the beginning stage, while the coefficient of Hispanic Americans was still significant at the post-peak stage. It can be evidence that minorities suffered a higher risk of transmission than the white only, and Hispanic Americans faced a worse situation. The focus on them may not be enough (Tucker, 2020). Besides, the relationships between rates of poverty and public transit commuting and case density were negative. It can be credited to lockdown policies narrowing travel areas to decrease the risk of transmission (Oraby et al., 2021). While the benefits of lockdown policies might decrease since the distance traveled became a significantly positive factor at the post-peak stage.

### GWR results

To check for spatial autocorrelation, the Moran Index was used to identify whether case density was randomly distributed in the study area. We found that COVID-19 case densities were clustered in all study regions, Harris and Fort Bend Counties (Moran’s I = 4.47, *p* < 0.05) at the beginning and (Moran’s I = 2.99, *p* < 0.05) at post-peak stages, Travis County (Moran’s I = 3.68, *p* < 0.05) at the beginning and (Moran’s I = 1.67, *p* < 0.05) post-peak stages, Clark County (Moran’s I = 0.176, *p* < 0.05) at the beginning and (Moran’s I = 0.146, *p* < 0.05) at post-peak stages, and NYC (Moran’s I = 0.647, *p* < 0.05) at the beginning and (Moran’s I = 0.146, p < 0.05) at post-peak stages.

**Fig. 3:**
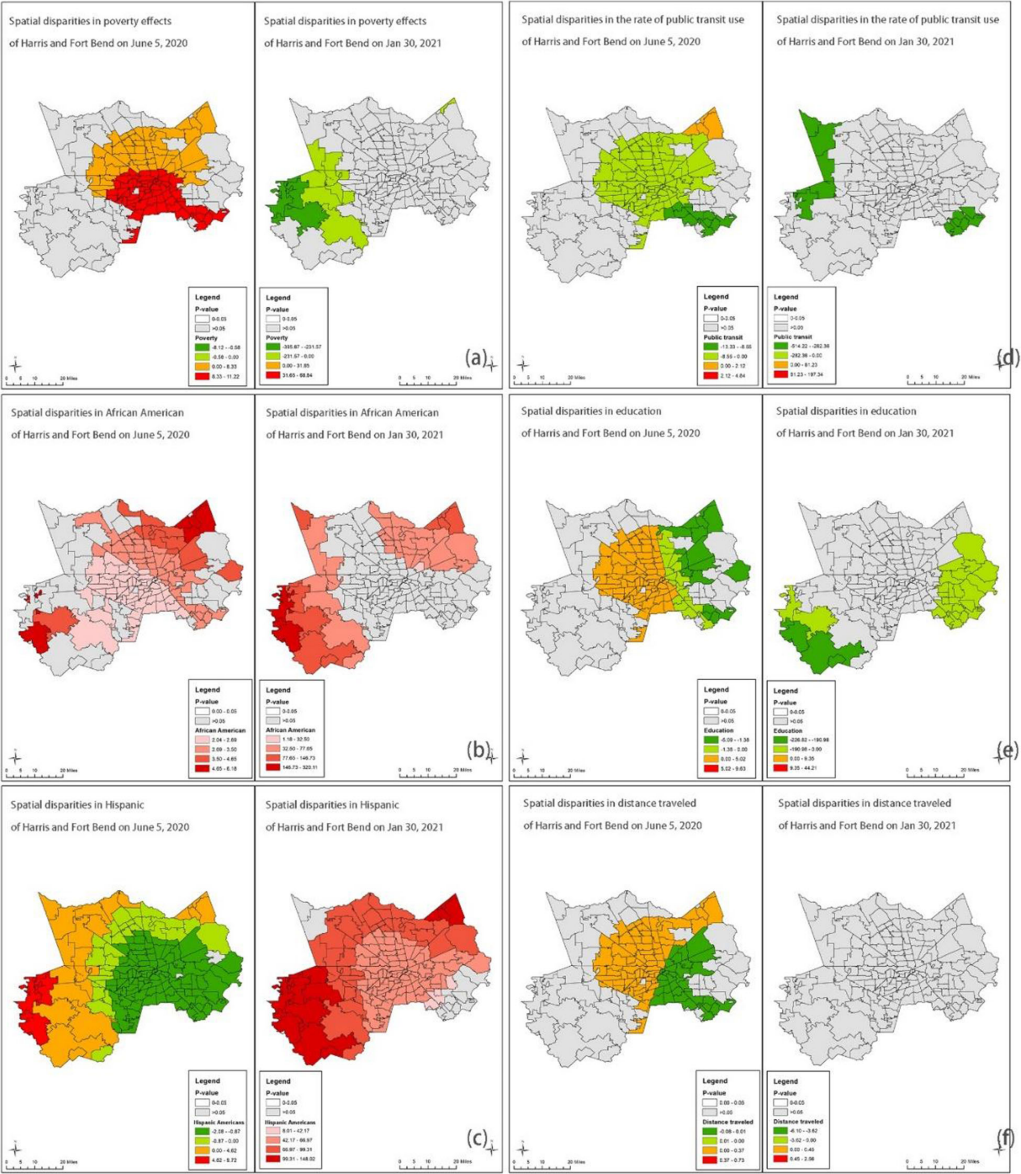
GWR results for Harris and Fort Bend Counties (Average R-square = 0.20 and 0.21) *Note: Early-stage data is taken on June 5, 2020, and post peak is taken on Jan 30, 2021*

**Fig. 4:**
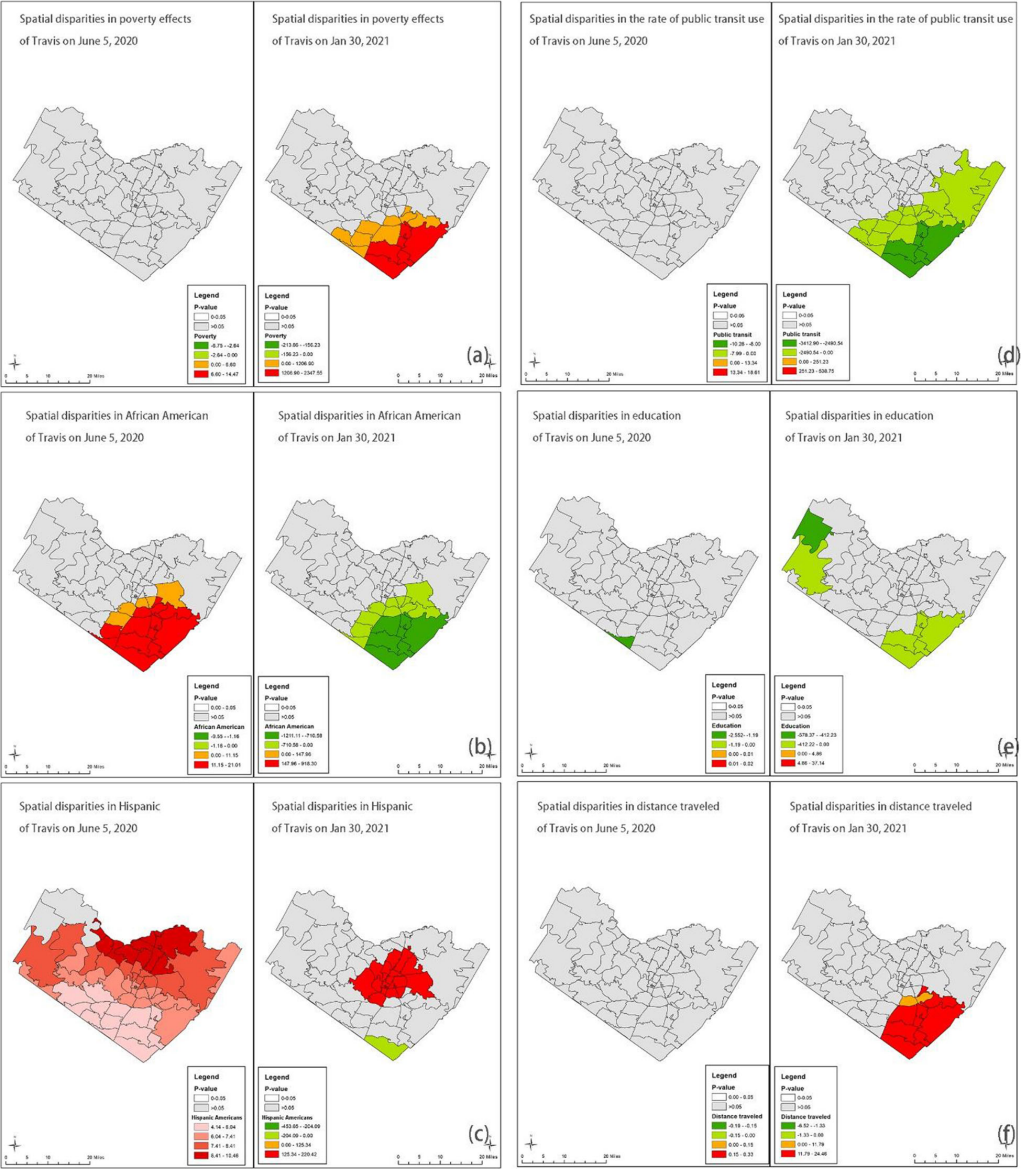
GWR results for Travis County (Average R-square = 0.49 and 0.34) *Note: Early-stage data is taken on June 5, 2020, and post peak is taken on Jan 30, 2021*

**Fig. 5:**
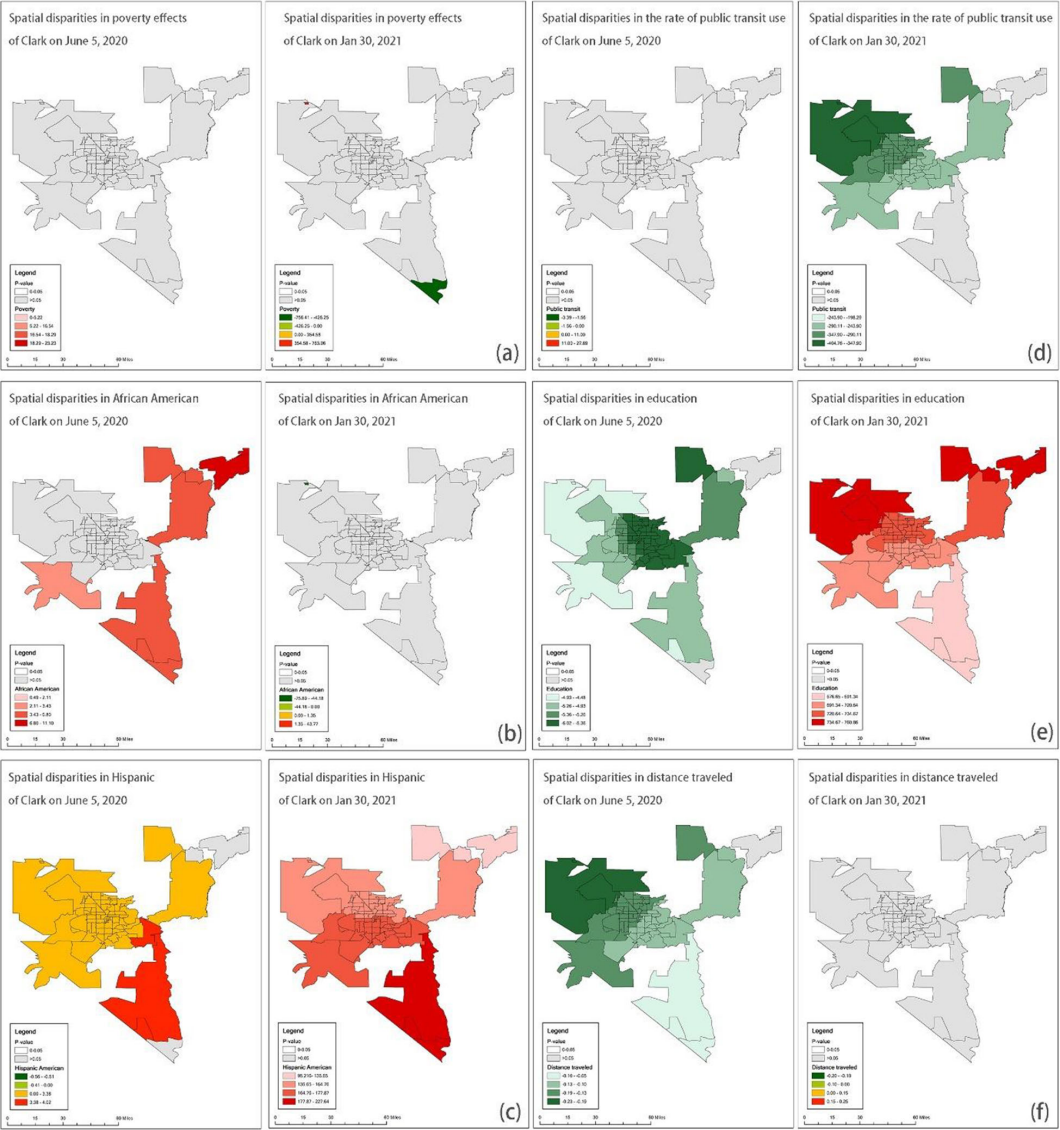
GWR results for Clark County (Average R-square = 0.76 and 0.89) *Note: Early-stage data is taken on June 5, 2020, and post peak is taken on Jan 30, 2021*

**Fig. 6:**
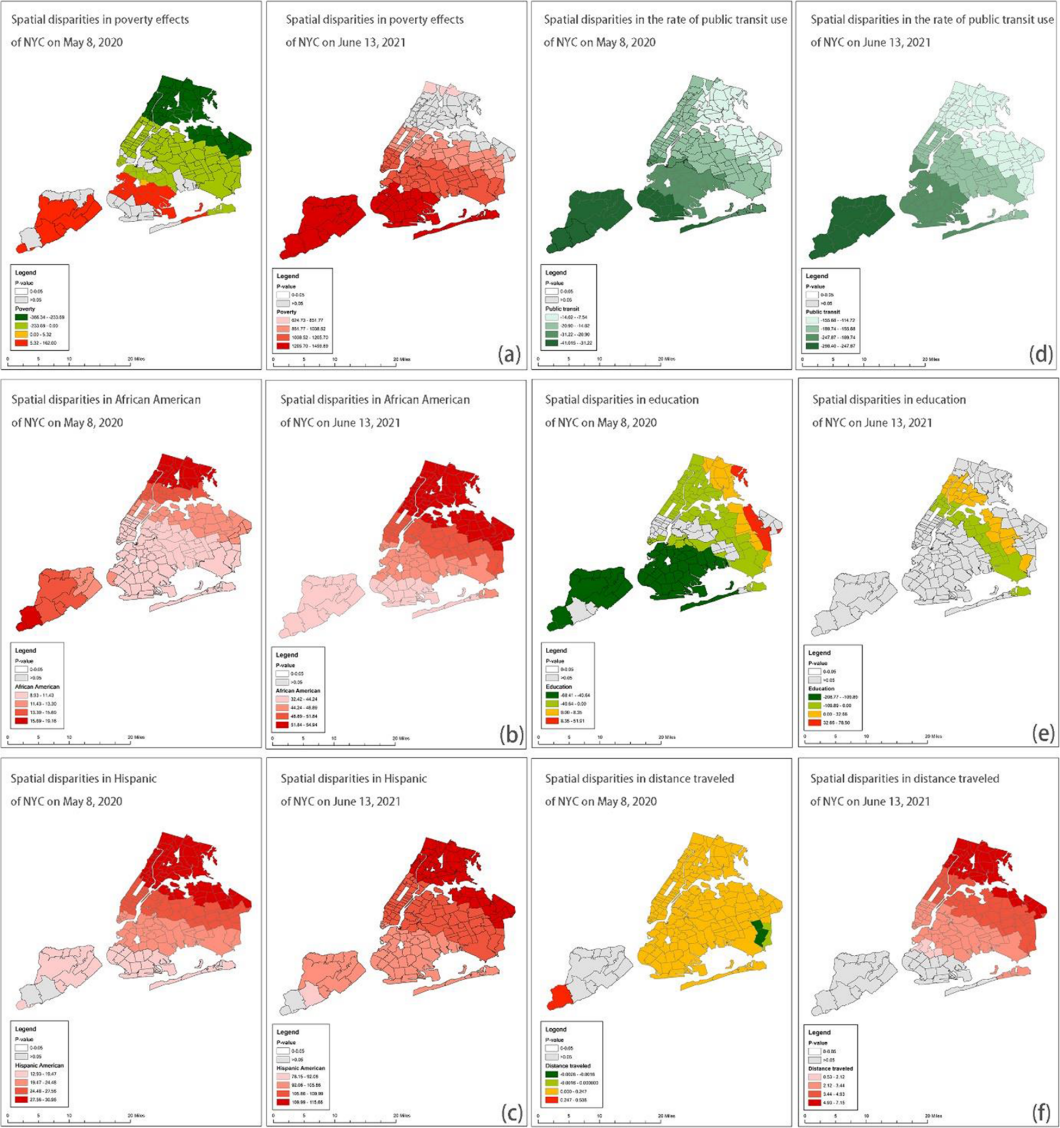
GWR results for NYC (Average R-square = 0.58 and 0.20) *Note: Early-stage data is taken on May 8, 2020, and post peak is taken on Dec 13, 2020*

Figures 3, 4, 5 and 6 show the GWR models. We calculated local *P*-values and dropped statistically insignificant areas (*p* > 0.05) of each explanatory variable. Figure 3 demonstrates the results of the GWR model in Harris and Fort Bend Counties. We found that households in poverty were likely at high risk of COVID-19 infection in the south of Houston at the beginning of the pandemic, while at the post-peak stage, the effect of households in poverty on COVID-19 case density was negatively correlated. People of color faced similar situations. For instance, African Americans and Hispanic Americans who live in the periphery of Harris and Fort Bend Counties were at higher risk of COVID-19 infection than those living in central areas both at the beginning and post-peak stages. Also, the public transit use might not be a critical factor in the spread of COVID-19 cases, neither at the beginning nor post-peak stages. Surprisingly, high-educated populations in the west of Houston might have been at higher risk of COVID-19 infection than those in other areas in the early stages. Figure 3 (f) shows that “active” residents, those who traveled a lot during the pandemic, might have been at high risk of COVID-19 infection at the beginning of the pandemic, but these travel behavior factors became insignificant at the post-peak stage.

Figure 4 presents the results of the GWR model in Travis County. Although the relationship between households in poverty and COVID-19 case density was insignificant at the beginning of the pandemic, this changed at the post-peak stage, with households in poverty experiencing a significantly high risk of COVID-19 infection. The way the pandemic in Travis County impacted racial and ethnic groups was different than in Harris and Fort Bend counties. At the beginning of the pandemic, African Americans in the southeastern part of Travis County were at high risk of COVID-19 infection, but this group appears to have fared better in the post-peak stage. Hispanic Americans living in the northern part of Travis County were at high risk of COVID-19 infection at the beginning of the pandemic. However, in the post-peak stage Hispanic Americans with high risk of infection were more concentrated to those living in downtown Austin. While education level did not significantly predict COVID-19 case density in Travis County, the travel behaviors factors may be worth noting. Figures 4 (d) and (f) demonstrate that although neither public transit use nor distance traveled were significantly related to COVID-19 case density at the beginning of the pandemic, residents with more distance traveled experienced a heightened risk COVID-19 infection at the post-peak stage.

Figure 5 presents the GWR results in a low-density area. In Clark County, poverty was not significantly correlated with COVID-19 infections since the relationship between households in poverty and case density was insignificant at the beginning and post-peak stages. Besides, the minorities who live in periphery areas have a higher risk of transmission than those who live in central Las Vegas. Also, the risks of transmission of minorities could be worse, especially of Hispanic Americans in the periphery. Figure 5 (d) indicates that communities with a high rate of public transit riders have low case density at the post-peak stage. Communities with higher rates of educated residents have lower case density at the beginning stage, but the relationship turned adverse at the post-peak stage. Surprisingly, the high median distance traveled did not mean a high risk of transmission in Clark County.

Lastly, Fig. 6 demonstrates the GWR result in NYC. We found that the relationship between poverty and infections did not change a lot. Households in poverty were likely at high risk of COVID-19 infection in the south of NYC at the beginning and the post-peak stages, while the relationships were negative. Look at the demographic factors in the north part where the rate of households in poverty was high. Considering the spatial patterns of demographic factors, we identified hotspots, Bronx and north of Queen for African Americans and Hispanic Americans during the pandemic. Besides, the median distance traveled was a positive factor at both stages, and the north part of NYC was the hotspot.

### RF results

**Fig. 7:**
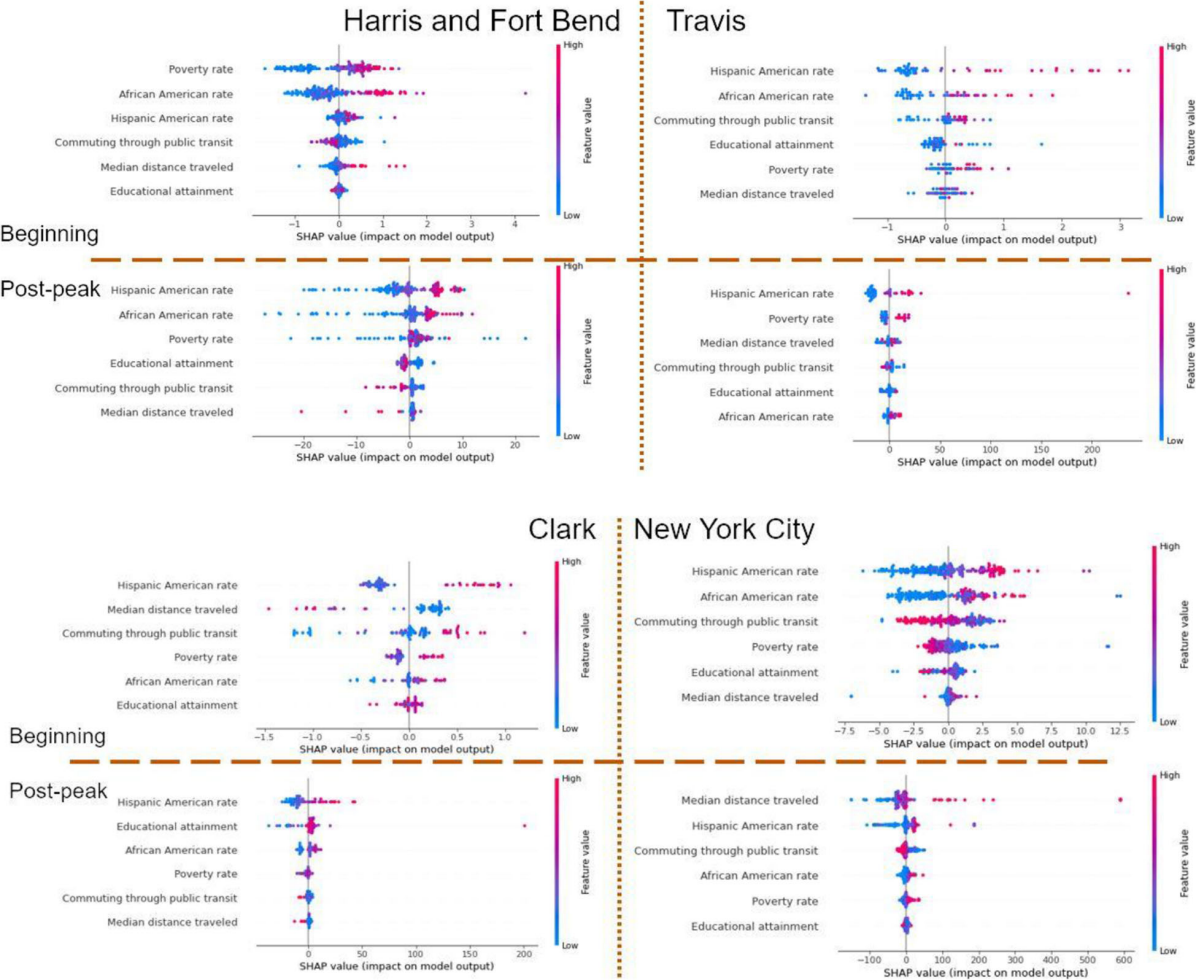
Distribution of each feature effects

To determine the parameter selections and reduce overfitting effects, we used grid searching and cross-validation in RF models. Once determining the parameter combination, we applied “SHAP” package to visualize the results of RF models (Fig. 7). In this figure, the variables (Y-axis) are ranked in descending order based on importance. We defined top three variables as the significant factors.

Focus on high-density areas. At the beginning stage, both demographic factors (African Americans rate and Hispanic Americans rate) were significant factors. It indicates that neighborhoods with a high rate of minorities were at a high risk of transmission. Communities with high rates of households in poverty were also at high risk, especially in Harris and Fort Ben counties. Besides, communities with high rates of households in poverty were also at high risk in both areas at the post-peak stage. The impacts of the Hispanic American rate remained positive in both regions, while the impacts of the African Americans rate changed, positive in Harris and Fort Bend but unclear in Travis County.

Look at the situation between low-density and the densest areas. Both at the beginning and post-peak stage, the impacts of Hispanic Americans factors were positive. In Clark, the other two significant factors were distance traveled and public transit usage at the beginning stage, while educational attainment and African Americans at the post-peak stage. In NYC, the significant factors were African American and public transit usage at the beginning stage, while distance traveled and public transit usage at the post-peak stage. Surprisingly, the poverty rate was not a significant factor in both samples.

### Result summary

We integrated the results, merging OLS and RF results and capturing the spatial distributions of impacts from GWR. If the variable in OLS or RF results was significant, it was marked as a significant variable. Then, the spatial patterns of these significant variables were captured. We defined two categories to demonstrate spatial patterns, center and periphery. The “center” means that the impacts of variables near downtown are more significant, correlated to more COVID-19 case density, while the “periphery” means that the situation in the periphery is worse.

Table 3 presents the integrated result focusing on demographic and economic variables. We marked the significant variables using the star sign. As it shows, the demographic disparities were significant in most cases. Neighborhoods with a high rate of minorities were likely to have severe COVID-19 infections. In most cases, minority neighborhoods in the periphery were likely facing worse situations except for the Hispanic neighborhoods in Travis.

**Table 3:** Summary of multiple-model results

	Harris and Fort Bend		Travis		Clark		NYC	
	Beginning	Post-peak	Beginning	Post-peak	Beginning	Post-peak	Beginning	Post-peak
Poverty	* center	* N/A		* periphery			* center	
African American	* periphery	* periphery	* periphery			* N/A	* periphery	
Hispanic American	* periphery	* periphery	* center	* center	* periphery	* periphery	* periphery	* periphery

Note: N/A means that the spatial patterns are unclear

Additionally, economic disparities are varied across cases. In Harris and Fort Bend and NYC, the neighborhoods with high rates of households in poverty were related to high COVID-19 case density, especially near downtown. Besides, the households in poverty located at the periphery faced even worse conditions. Different from the other samples, the households in poverty had severe infections at the post-peak stage than other neighborhoods in Travis.

## Discussion

COVID-19 has been a pandemic for around two years, impacting people’s daily lives around the world. With more and more people vaccinated, the daily confirmed cases are decreasing, and the symptoms are not as dangerous as what it was one year ago. It can be the dawn for the whole world to go back to normal lives, but the reliefs after the pandemic are worth being considered by policymakers. It is essential to understand demographic and economic disparities in the infections. Previous studies proved that racial disparities in COVID-19 infections, hospitalizations, and mortalities were significant, while they disproportionally focused on the big and dense cities and used low-resolution data. Also, due to the data limitation, previous studies were based on cross-sectional data through different methods, but there lacks a comparison between the situation at the beginning and later and compare results from these models.

To fill the research gaps, we took four regions as the study area and applied three different types of methods to measure the demographic and economic disparities and compared the changes in disparities at two pandemic periods. Results indicate that demographic and economic disparities were significant, but their significance changed across cities and periods. In Harris and Fort Bend counties, the COVID-19 infections were serious in neighborhoods with high rates of households in poverty, African Americans, and Hispanic Americans, both at the beginning and post-peak stages. Besides, the neighborhoods with a high poverty rate near city centers and minorities gathering in the periphery were hotspots. In Travis County, the Hispanic American neighborhoods suffered severe infections, especially near downtown. African American neighborhoods were hotspots at the beginning stage, while the poor households turned to at high risk of infections at the post-peak stage. Similarly, Hispanic American neighborhoods were at high risk of infections at both stages in Clark County, and these neighborhoods in the periphery faced even severe conditions. Also, the case density was likely high in neighborhoods with a high rate of African Americans at the post-peak stage. Last, demographic and economic disparities in NYC were significant. Households in poverty living in downtown and Brooklyn and minorities who lived in the periphery were the most vulnerable groups at the beginning stage, while these minorities, especially Hispanic neighborhoods, were still at high risk.

We acknowledge limitations in this study. First, the limitation in data is significant. Since most regions only published the recent data and did not provide historical data, we had to spend longer than expected time on data collection. We planned to use rates of positive COVID-19 cases as the outcome variable, which are commonly used in previous studies (Liu et al., 2020a; Wu & Zhang, 2021), but only NYC provided this data. So, we chose case densities as the alternative, which was used in some studies (Cousins, 2020; Yellow Horse et al., 2021). If there is extra available data in the future, future studies can compare the changes when choosing different outcome variables. Also, this study investigates the demographic and economic disparities of four regions. It would be better if extra cases were introduced.

Additionally, this study only focused on two minorities, African Americans and Hispanic Americans, since they are the two minorities with a high portion of the total population, while focusing on two minorities may not be enough. We notice that other minorities also suffered more serve covid-19 infections and hospitalization than the white only (Keating et al., 2020; Qeadan et al., 2021). Future studies holistically investigate the racial disparities to help policymakers allocate potential economic reliefs and health services from a holistically perspective.

Third, the mismatches in defining begging and post-peak stages can be critical. We roughly defined the beginning stage as of May 8, 2020, and the post-peak stage as of June 13, 2021, in NYC, while this definition was different from the others. Future research should focus on defining the periods since they may vary across regions since the first case, peak, and trough of COVID-19 infections were different across cities to get reliable and robust results.

Albeit these limitations, several research and policy implications are worth noting. First, the significant signs of household in poverty in Travis County and rate of African American in Clark County change from at the beginning stage to the post-peak stage. One hypothesis is that the households in poverty in Travis County and African Americans in Clark County were hard or refused to get the test at the beginning stage when medical resources were overwhelmed (AMA, 2020; Gray, 2021). So, the number of cases was underestimated. This finding echoes evidence from local reports (Bernier, 2019; Johnson, 2021). Future studies should investigate medical accessibility affects the COVID-19 infections when data is available.

Second, we encourage local governments and agencies to take action for vulnerable populations during the recovery period of the pandemic. This study shows that households in poverty and people of color experienced a heightened risk of COVID-19 infection, supporting the claim that the demographic and economic disparities are significant in the U.S. (Henry, 2020; Rozenfeld et al., 2020; Zamarripa & Roque, 2021). We further identify the spatial patterns of these disparities. Households in poverty near downtown and minority neighborhoods at the periphery are at risk of infections in most cases, but it is worth noting that these spatial patterns may change. Hence, policymakers need to consider the recovery plan based on local conditions (OECD, 2020).

Governments have provided massive recovery plans to support the vulnerable population during and after the pandemic. Rent relief programs and other economic reliefs are important parts of the recovery plans (Clark Housing Assistance Program, n.d.; Houston-Galveston Area Council, n.d.; NY HCR, n.d.; Texas Rent Relief Program, n.d.). Although there are rent relief programs in all study cases, it is worth noting that these plans do not have focuses, while according to this study, vulnerable groups in special areas suffer more serve infections than the vulnerable in other areas. We suggest that the local government launch a step-by-step rent relief program under the premise of fairness. In Harris and Fort Bent counties, according to a report on January 2021, the minorities are under high risk of eviction crisis (Bustamante, 2021). The communities, local governments, and non-government organizations need quick actions and preferentially help the poverty households in south Houston and minority neighborhoods in the west of Fort Bend. Not surprisingly, these neighborhoods that need to be preferentially considered are where vulnerable populations gather (Olin, 2020).

Considering the distribution of historically vulnerable groups in Travis County, the eastern side of the county should be prioritized in public resource allocation. Besides, around 35% of the population in Travis County identify as Hispanic. It is in line with what policymakers in the Travis have found, and local, and state governments are currently addressing these disparate COVID-19 issues in East Austin (DuPree, 2020). We suggest that the local government continues to pay attention to how people of color in these areas are affected by the pandemic and, more broadly, how they are impacted in terms of disasters and public health. Besides, the Hispanic American neighborhoods in the north of Travis are at high risk of infections. We call collaborations between communities, local governments, and private companies to help the Hispanic renters avoid of evictions.

Situations in Clark County are different with the above cases. At the post-peak stage, Hispanic neighborhoods on the south part are at high risk of infestations. Since this area is low-density, we assume that the renter rate is not high, so the households may not support the rent relief program. We suggest that communities visit neighborhoods, survey whether they are facing a fiscal crisis, and help them if needed. Besides, considering Las Vegas reopening, although the mask mandate is ongoing, the breakthrough risks for workers are high (Hynes & Bergin, 2021). We recommend local government help workers get free tests frequently and allocate budgets on health facilities, like quick-testing hubs.

In NYC, we identify hotspots where vulnerable populations face serves infections. The local communities need to focus on poverty households in Staten Island and Brooklyn, African American neighborhoods in the Bronx, and Hispanic neighborhoods in the Bronx and north of Queens, while these areas are vulnerable regions (NYC Health, 2013). The NYC rent and economic relief program has noticed these areas and allocated extra supports to the restaurants/stores by minorities (NYC, 2020). Though NYC has done well in supporting vulnerable populations, how these relief programs work needs further exploration.

Besides these practical implications, our study finds significant changes in demographic and economic disparities in COVID-19 infections across regions, and densities may not be the cause of these changes comparing the results in Harris and Fort Bend counties and NYC. Previous studies also pointed out these differences, but few of them clearly claim the reasons for these changes (Figueroa et al., 2020; Jiao & Azimian, 2021; Mackey et al., 2021). We encourage future research to explore the reasons for different demographic and economic disparities in COVID-19 infections.

Fourth, by comparing the performance of these types of models (Table 4), the OLS models performed worst in most cases. GWR models could reflect spatial heterogeneity, but their performance is not as good as RF models. Considering the increase rate in R-square, we noticed that the RF model could have a much better performance when there are more observations. In other words, in dealing with big data analysis, the RF model can perform better than OLS and GWR. However, when the number of observations is small, the RF model may not be better than OLS even. We encourage future researchers to combine the GWR and RF, introducing the spatial analysis into machine learning (Al-Abadi & Shahid, 2016; Hu et al., 2021).

**Table 4:** Model performance based on R-square value

		OLS	GWR	RF
Beginning stage	Harris and Fort Bend	0.20	0.20	0.72^a^
	Travis	0.49	0.49	0.90^a^
	Clark	0.65	0.76	0.81^a^
	NYC	0.28	0.58	0.91^a^
Post-peak stage	Harris and Fort Bend	0.15	0.21	0.40^a^
	Travis	0.33	0.34	0.71^a^
	Clark	0.86	0.89^a^	0.85
	NYC	0.41	0.20	0.92^a^

Note. ^a^ means the better performance model

## Conclusion

This study observed the demographic and economic disparities in COVID-19 infections at the beginning and post-peak stages, choosing four U.S. areas as the empirical cases. The results indicate significant disparities and encourage more attention on households in poverty near city centers and minority neighborhoods in the periphery. With vaccines taken, outbreaks have slowed down, and people are eager to go back to normal lives. As the dawn of ending this pandemic appears, it is worth considering how to recover from this disaster. Suggestions in this study show potential suggestions that can be used for policymakers to allocate recovery plans and economic/rent reliefs during the post-pandemic stage.

## Data Availability

N/A
